# PEG-Derivatized Dual-Functional Nanomicelles for Improved Cancer Therapy

**DOI:** 10.3389/fphar.2019.00808

**Published:** 2019-07-19

**Authors:** Yanping Li, Ting Zhang, Qinhui Liu, Jinhan He

**Affiliations:** ^1^Laboratory of Clinical Pharmacy and Adverse Drug Reaction, West China Hospital of Sichuan University, Chengdu, China; ^2^Department of Pharmacy, West China Hospital of Sichuan University, Chengdu, China

**Keywords:** polyethylene glycol (PEG), dual-functional nanomicelles, cancer therapy, multidrug resistance, drug loading capacity, cancer immunochemotherapy

## Abstract

Polymeric micelles have attracted considerable attention for effective delivery of poorly water-soluble cancer drugs. Polyethylene glycol (PEG), which has been approved for human use by the US Food and Drug Administration, is the most commonly used hydrophilic component of polymeric micelles because it is biocompatible and biodegradable. One disadvantage of traditional polymeric micelles is that they include a large amount of inert carrier materials, which do not contribute to therapeutic activity but increase cost and toxicity risk. A better alternative may be “dual-functional” micellar carriers, in which the hydrophobic carrier material (conjugated to PEG) has intrinsic therapeutic activity that complements, or even synergizes with, the antitumor activity of the drug cargo. This review summarizes recent progress in the development of PEG-derivatized dual-functional nanomicelles and surveys the evidence of their feasibility and promise for cancer therapy.

## Introduction

Cancer has become the “first killer” that threatens human health (Siegel et al., 2017; Smith et al., 2017; Torre et al., 2017). Chemotherapy, one of the most important tools in clinical cancer treatment, inhibits growth of primary tumors and suppresses proliferation of metastatic tumor cells (Chabner and Roberts, 2005). Although many anticancer drugs have been approved, their clinical application is often compromised by poor water solubility, short circulation time, and serious systemic side effects (Shi et al., 2016; Youn and Bae, 2018). Nanomaterials have been widely used in cancer therapy (Wicki et al., 2015). Various nanocarriers, including nanoparticles (Geszke-Moritz and Moritz, 2016), liposomes (Cheng and Ji, 2019), micelles (Cagel et al., 2017), and dendrimers (Ray et al., 2018), have opened up new possibilities for sustained, controlled, and targeted cancer drug delivery, achieving higher effective drug concentrations at the tumor site with fewer off-target side effects. In particular, polymeric micelles have attracted attention as multifunctional drug delivery systems for poorly water-soluble agents (Zhang et al., 2014h; Biswas et al., 2016).

**Figure 1:**
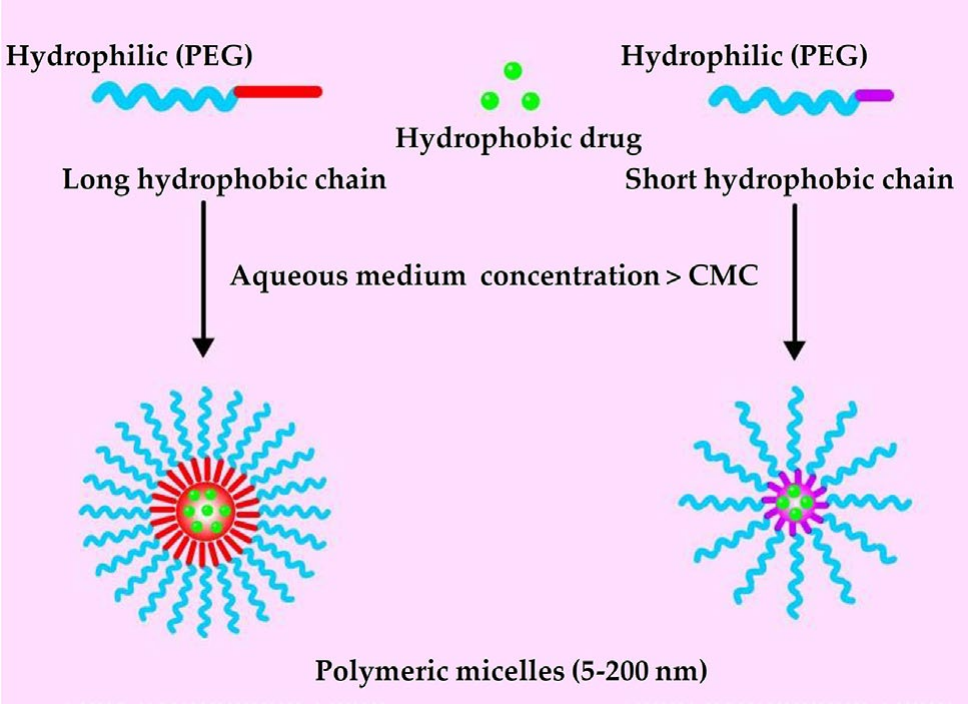
General structure of polymeric micelles loaded with hydrophobic drug.

Polymeric micelles are self-assembling nanoparticles (5–200 nm) composed of amphiphilic polymers. The structure of polymeric micelles follows and exemplifies the similar structure of micelles proposed as per different micellar theories (Gothwal et al., 2016). It is composed of a “core,” which is usually a hydrophobic section while the exterior, which is also known as “shell,” represents a hydrophilic block of the copolymer structure (**Figure 1**) (Mohamed et al., 2014; Zhang et al., 2014d). Polymeric micelles are easy to prepare, highly biocompatible, and quite efficient at encapsulating and delivering drugs in the body (Gong et al., 2012). Polymeric micelles can encapsulate water-insoluble agents in their hydrophobic core, improving bioavailability (Kedar et al., 2010; Tanbour et al., 2016). A hydrophilic shell of polyethylene glycol (PEG) can significantly prolong the circulation time of polymeric micelles in the blood (Suk et al., 2016). Polymeric micelle in the 10–100 nm range can pass through the leaky vasculature and passively accumulate in tumors *via* the enhanced permeability and retention effect (EPR) (Maeda et al., 2009; Fang et al., 2011; Maeda et al., 2013).

PEG, which has been approved by the US Food and Drug Administration (FDA) for use in human, is the most commonly used hydrophilic component of polymeric micelles (Molineux, 2002; Duncan, 2014; Qu et al., 2018). It is biodegradable and biocompatible because it does not form toxic metabolites, and it is commercially available in molecular weights ranging from 500 to 20,000 Da. Surface modification of PEG-based nanocarriers can reduce interactions with plasma proteins and nonspecific uptake by the reticuloendothelial system, prolonging time in systemic circulation (Bae and Kataoka, 2009; Wang et al., 2018a). The multifunctionality of PEG allows multiple drug or targeting molecules to be conjugated to the same polymer chain. Currently, several PEG-derivatized polymeric micelles carrying anti-cancer drugs are under clinical evaluation, including NK012, NK105, SP1049C, NK911, and Genexol-PM (Cabral and Kataoka, 2014; Mohamed et al., 2014; Deshmukh et al., 2017). In fact, regulatory authorities in South Korea have approved Genexol-PM for use against breast cancer.

In the “hydrophilic shell-hydrophobic core” architecture of polymeric micelles, the hydrophobic core provides a loading space for poorly water-soluble drugs (Zhang et al., 2014d). The polymers most commonly used to build the hydrophobic core are polyesters and polyamides, such as polycaprolactone, poly(lactic acid), poly(glycolic acid), and poly(l-histidine) (Zhang and Zhuo, 2005; Zhang et al., 2014a; Zhang et al., 2014c). Most of these hydrophobic components are inert carrier materials without therapeutic activity. In other words, they add to the cost and toxicity risk of the drug delivery system, without contributing to therapeutic efficacy (Qu et al., 2017b). This has led researchers to explore nanocarriers that themselves have therapeutic effects.

**Figure 2:**
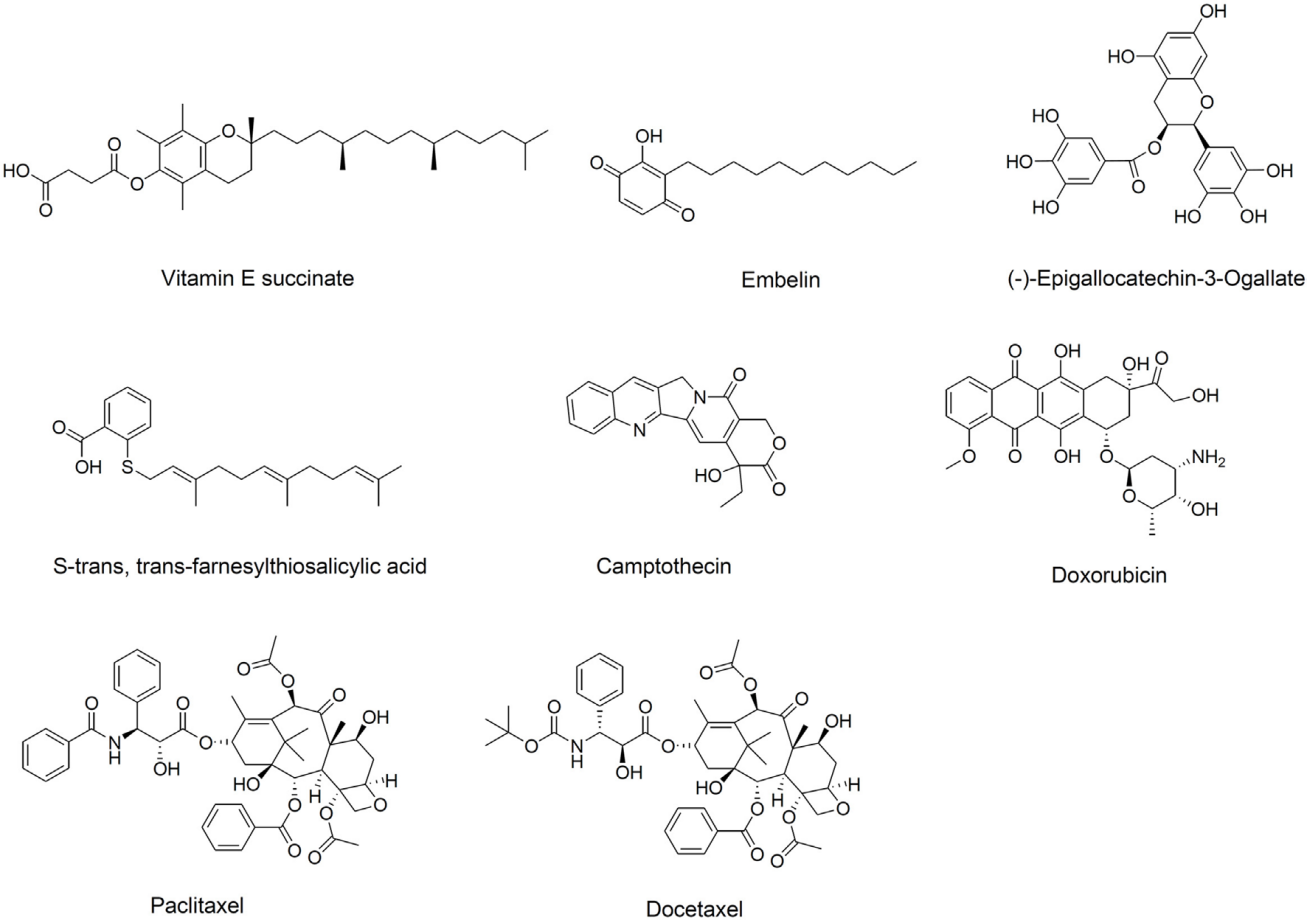
Chemical structure of hydrophobic domains in polyethylene glycol (PEG)-derivatized dual-functional micellar carriers.

Since Ringsdorf proposed the concept of “polymeric prodrug” in 1975, the utility of polymer-drug conjugates in clinical therapy has been well demonstrated (Hoste et al., 2004; Ringsdorf, 2010). Interestingly, hydrophobic drugs conjugated to hydrophilic polymers can self-assemble into micelles, which can then be loaded with another therapeutic molecule (Ulbrich and Subr, 2004; Greco and Vicent, 2008). Numerous bioactive hydrophobic agents have been conjugated to PEG to form so-called “dual-functional” micellar carriers, which have intrinsic antitumor activity and that deliver anti-tumor drug cargo (Mi et al., 2011; Huang et al., 2012; Chung et al., 2014; Zhang et al., 2014e). PEG-derivatized dual-functional micelles are typically prepared with one of two types of hydrophobic material (Qu et al., 2017b): anti-cancer natural products not yet used clinically, such as vitamin E succinate, (−)-epigallocatechin-3-gallate, embelin, and S-trans,trans-farnesylthiosalicylic acid, or anti-cancer molecules already established in the clinic, such as camptothecin (CPT), doxorubicin (DOX), paclitaxel (PTX), and docetaxel (**Figure 2**).

In this review, we summarize recent progress in PEG-derivatized dual-functional micellar carriers and their application in cancer therapy. Strategies to improve carrier–drug interactions are discussed because two challenges in the development of polymeric micelles are their drug-loading capacity and stability. Recent developments in exploiting dual-functional carriers for therapies combining immuno- and chemotherapy are also surveyed.

## PEG-Derivatized Dual-Functional Micelles With Intrinsic Anti-Cancer Activity

### PEG-Derivatized Vitamin E

Vitamin E succinate (D-α-tocopherol, α-TOS) is a derivative of vitamin E that shows activity against various types of cancers, including breast, lung, and prostate (Malafa and Neitzel, 2000; Malafa et al., 2002; Kline et al., 2004). α-TOS can selectively kill tumor cells without affecting normal cells by inhibiting angiogenesis, suppressing activation of NF-κB, and generating reactive oxygen species (Dong et al., 2007; Jin et al., 2014). It can also enhance the cytotoxicity of PTX and DOX (Kanai et al., 2010), and it can inhibit P-glycoprotein (P-gp) to overcome multidrug resistance (MDR) (Constantinides et al., 2006).

The poor water solubility of α-TOS has limited its use *in vivo*, leading researchers to explore possibilities with D-α-tocopheryl PEG succinate (vitamin E TPGS, or simply TPGS), which is a water-soluble derivative of α-TOS generated by esterification with PEG (Youk et al., 2005). TPGS acts as a nonionic surfactant: it exhibits amphipathic properties and can form micelles in aqueous media at a critical micelle concentration (CMC) of 0.2 mg/ml. It has been investigated as an absorption enhancer, emulsifier, solubilizer, additive and permeation enhancer, and stabilizer (Zhang et al., 2012; Guo et al., 2013). TPGS also inhibits P-gp-mediated drug efflux by inhibiting the ATPase of P-gp and mitochondrial respiratory complex II (Dong et al., 2008; Collnot et al., 2010). The relatively high CMC of TPGS and its relatively short PEG chains mean that TPGS-based micelles usually leave the circulation quickly and accumulate in the liver and spleen (Tan et al., 2017; Yang et al., 2018a). Therefore, TPGS is usually mixed with other lipids or copolymers to form micelles.

**Figure 3:**
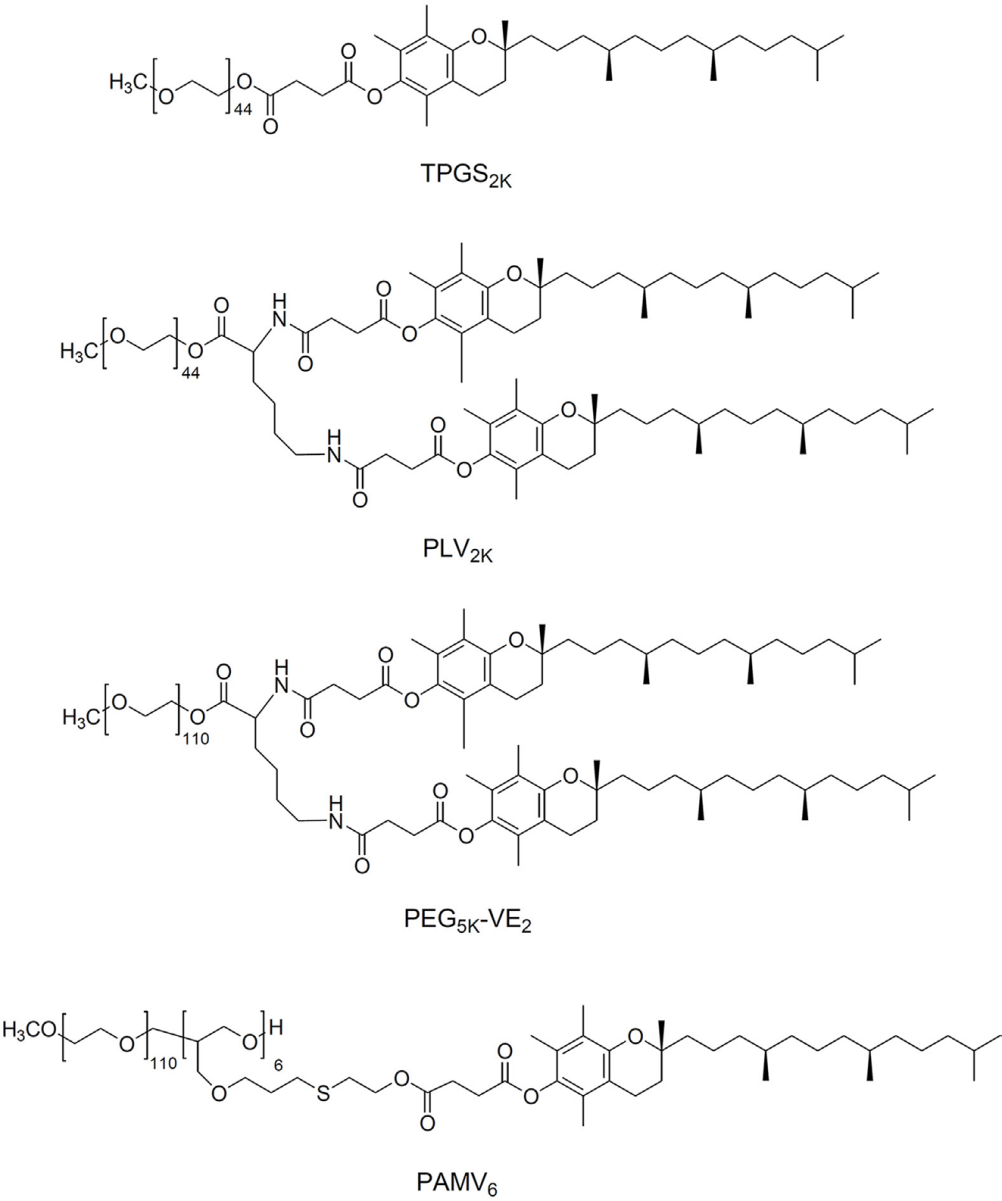
Chemical structure of TPGS derivatives.

Research groups have taken different approaches to improving the properties of TPGS as a micelle carrier (**Figure 3**). Conjugating tocopheryl succinate to PEG 2000 leads to TPGS_2K_, which has a much lower CMC than TPGS, allowing formation of stable drug-loaded micelles without addition of other lipids or polymers. For example, one group developed TPGS_2K_ polymer-based micelles with a CMC of only 0.0219 mg/ml (Mi et al., 2011). Docetaxel was successfully loaded in TPGS_2K_ micelles to form a uniform sphere with an average diameter of 15 nm. *In vitro* antitumor results showed that docetaxel-loaded TPGS2K micelles dramatically decreased the IC_50_ values of MCF-7 cells (0.526 g/ml) compared with that of the free docetaxel (103.4 g/ml), indicating a synergistic therapeutic effect between TPGS_2K_ and docetaxel. Another group conjugated PEG to two vitamin E molecules to form star-shaped copolymer PLV_2K_ micelles with a CMC of only 1.14 μg/ml (Wang et al., 2012). DOX was encapsulated into the hydrophobic core of PLV_2K_ with encapsulation efficiency as high as 94.5%, and the drug-loaded micelles inhibited growth of 4T1 breast cancer tumors in mice more effectively than free DOX or DOX-loaded in TPGS.

Screening two PEG lengths (PEG_2K_ or PEG_5K_) and two molar ratios of PEG to vitamin E (1:1 or 1:2) identified PEG_5K_-VE_2_ micelles comprising one PEG_5K_ and two vitamin E molecules as having the lowest CMC and most efficient PTX loading (Lu et al., 2013a; Lu et al., 2014c). Loading PTX into PEG_5K_-VE_2_ inhibited tumor growth in mice significantly better than loading it into PEG_2K_-VE or PEG_5K_-VE_2_, or than using Taxol. Our group has analyzed how the molar ratio of PEG to vitamin E affects the stability, drug loading capacity, and tumor targeting efficiency of TPGS-based micelles *in vitro* and *in vivo* (Li et al., 2016a; Li et al., 2016b). PAMV_6_, which is formulated with a 1:6 molar ratio of PEG to vitamin E and shows a wheat-like structure, showed the lowest CMC, greatest stability in serum, and highest tumor distribution and retention. Moreover, PAMV_6_ inhibits P-gp efflux activity, not by affecting P-gp expression, but by decreasing mitochondrial membrane potential and intracellular levels of ATP (Li et al., 2016a). As a result, pirarubicin-loaded PAMV_6_ micelles inhibited the growth of MCF-7/ADR xenograft tumors in mice significantly better than free pirarubicin, with significantly less systemic and organ-specific toxicity.

### PEG-Derivatized EGCG

The most abundant catechin in green tea, (−)-epigallocatechin-3-gallate (EGCG), shows activity against cancer, diabetes, neurodegenerative disease, stroke, obesity, and other disorders (Chakrawarti et al., 2016; Song et al., 2017). EGCG can inhibit tumor growth and metastasis by interrupting key signaling and metabolic pathways that support cancer cell survival without damaging normal cells (Shankar et al., 2007; Wang et al., 2018b). For example, EGCG can down-regulate PEA15 expression and enhance TRAIL-mediated apoptosis in tumor cells (Siegelin et al., 2008; Le et al., 2018). EGCG interacts with the estrogen receptor to inhibit estrogen-induced proliferation of breast cancer cells and sensitize hormone-responsive tumors to drugs such as tamoxifen that target steroid receptors (Farabegoli et al., 2007; Tu et al., 2011).

Despite its clear therapeutic effects, EGCG remains far from clinical use, in large part because of its low stability and bioavailability. The several hydroxyl groups in EGCG make it highly hydrophilic, which resulted in short circulation half-time. One group developed a micellar nanocomplex (MNC) comprising two EGCG derivatives that bind to proteins in a spatially ordered structure (Chung et al., 2014). MNCs self-assemble in two steps: first, oligomerized EGCG (OEGCG) complexes with the humanized monoclonal therapeutic antibody Herceptin (trastuzumab) to form a core; second, PEG-EGCG assembles as a shell around this core. The resulting MNC is monodisperse and spherical, with a mean diameter of about 90 nm and good stability in serum at 37°C for 15 days. Both *in vitro* and *in vivo* therapeutic studies showed that Herceptin-MNC had superior anti-tumor effects to free Herceptin. While Herceptin on its own shows anti-tumor activity, it synergizes with the EGCG within MNC to cause even greater inhibition, with a combination index of 0.93. Similarly, replacing Herceptin with interferon α-2a generates an MNC that inhibits the growth of HAK-1B human liver cancer cells more than free interferon α-2a, with a combination index of 0.46.

EGCG inhibits P-gp (Jodoin et al., 2002) and can sensitize drug-resistant tumor cells to DOX and increase DOX accumulation in MDR cells (Liang et al., 2010; Song et al., 2017). Thus, combination therapy with EGCG and DOX may simultaneously overcome DOX-induced cardiotoxicity and MDR. Special methods are needed to co-load DOX and EGCG into micelles, since DOX is hydrophobic while EGCG is hydrophilic and unstable. To achieve such co-loading, a novel green tea catechin-based polyion complex was developed in which DOX and EGCG were co-loaded *via* electrostatic interactions as well as interactions between EGCG and poly(ethylene glycol)-block-poly(lysine-co-lysine-phenylboronic acid) (Cheng et al., 2016). This allowed DOX to be loaded into the micelles with a drug loading capacity of 12.4% and drug loading efficiency of 55%. The ability of EGCG to scavenge oxygen free radicals and inhibit P-gp means that these vesicles may simultaneously overcome DOX-induced cardiotoxicity and MDR (Mei et al., 2004; Yao et al., 2017).

### PEG-Derivatized Embelin

Embelin (2,5-dihydroxy-3-undecyl-1,4-benzoquinone), a major active constituent of the *Embelia ribes* Burn plant, possesses analgesic, anti-diabetic, anti-inflammatory, and hepatoprotective activities (Chitra et al., 1994; Naik et al., 2013; Peng et al., 2014). It also inhibits growth of breast, colon, prostate, and pancreatic tumors *via* several different mechanisms. In most cases, the compound induces intrinsic or extrinsic apoptotic cell death and modulates signaling pathways involving NF-κB, p53, PI3K/AKT, and STAT3 (Heo et al., 2011; Kim et al., 2013; Ko et al., 2018). Interestingly, *in silico* screening predicts that embelin inhibits the X-linked inhibitor of apoptosis protein (XIAP), which is over-expressed in a variety of cancers, especially drug-resistant ones (Nikolovska-Coleska et al., 2004; Hussain et al., 2017). Several synthetic peptide inhibitors of XIAP have been reported, and some are in clinical trials, but embelin would be the only naturally occurring non-peptide XIAP inhibitor reported so far.

Embelin is poorly water-soluble and has limited oral bioavailability. Modifying it with PEG can significantly increase its water solubility, and the resulting PEG-embelin conjugates can self-assemble to form micelles and efficiently encapsulate hydrophobic drugs such as PTX and DOX (Huang et al., 2012). Screening two PEG lengths (PEG_3.5K_ or PEG_5K_) and two molar ratios of PEG to EB (1:1 or 1:2) showed that PEG_5K_-EB_2_, in which two embelin molecules were linked *via* aspartic acid to one PEG_5K_, showed the lowest CMC and highest PTX loading efficiency (Lu et al., 2013b). When these micelles were loaded with PTX, the embelin and PTX synergized to inhibit cancer cell growth *in vitro* even when PTX were present at nanomolar concentrations. The same micelles also inhibited growth of 4T1 breast cancer tumors and PC-3 prostate cancer tumors in mice better than Taxol, with minimal toxicity.

To improve the targeting ability of PEG_5K_-EB_2_ micelles, folic acid (FA) was coupled to the surface and the resulting FA-PEG_5K_-EB_2_ were able to readily self-assemble to form nanomicelles with a particle diameter around 20 nm (Lu et al., 2014b). FA-PEG_5K_-EB_2_ significantly facilitated the intracellular uptake of DOX over free DOX and Doxil in breast cancer cells 4T1.2, and drug-resistant cells NCI/ADR-RES. Consistent with better tumor targeting, the FA-decorated micelles inhibited the growth of 4T1.2 metastatic breast cancer tumors in mice significantly more than free DOX, Doxil or the micelles without FA, and with lower toxicity.

PEG-embelin micelles, in addition to enhancing the anti-tumor efficacy of PTX or DOX, can also inhibit P-gp ATPase activity analogously to TPGS and thereby can sensitize drug-resistant NCI/ADR-RES cells to DOX (Lu et al., 2013b; Lu et al., 2014b). Further studies should clarify whether PEG-embelin micelles act as a non-competitive or competitive inhibitor of the P-gp ATPase.

### PEG-Derivatized Farnesylthiosalicylic Acid

*S-trans,trans*-farnesylthiosalicylic acid (FTS) is a synthetic farnesylcysteine mimetic with strong potential to be an effective cancer drug (Kloog and Cox, 2000; Erlich et al., 2006). As a first-in-class Ras inhibitor, it is in phase II clinical trials to treat pancreatic cancer and lung adenocarcinoma (Riely et al., 2011; Laheru et al., 2012). FTS may also be effective against other cancers in which mutated Ras proteins are constitutively active (Simanshu et al., 2017), and it so far has shown minimal side effects (Gana-Weisz et al., 2002). FTS can also suppress cancer cell growth and proliferation by inhibiting mTOR, especially in endocrine-resistant breast cancer (Mcmahon et al., 2005).

**Figure 4:**
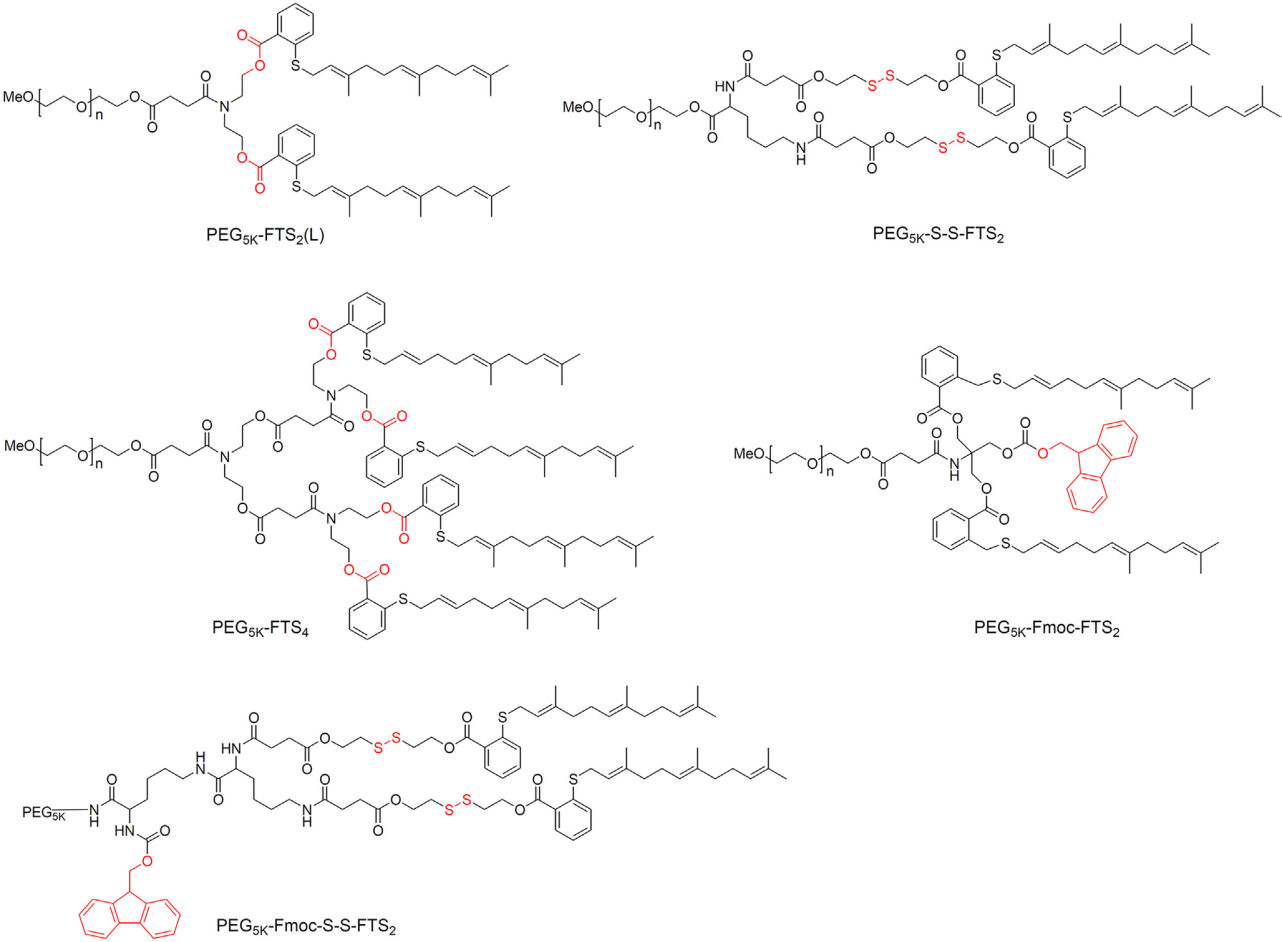
Chemical structure of PEG-FTS conjugates.

Its long hydrophobic chains make FTS poorly water-soluble, so it is usually PEGylated when forming micelles (**Figure 4**). For example, conjugation two FTS to PEG *via* a labile ester linkage readily forms PEG_5K_-FTS_2_(L) micelles in aqueous solution, which inhibit tumor growth in mice to a similar extent as free FTS (Zhang et al., 2013). Loading these micelles with PTX or curcumin led to synergy between these compounds and the FTS, such that micelle cytotoxicity was higher than with PTX or curcumin on their own (Zhang et al., 2013; Chen et al., 2014). Breaking of the linkage between PEG and FTS appears to be critical for its anti-tumor activity: replacing the ester linkage with a more stable amide linkage resulted in significantly lower cytotoxicity (Zhang et al., 2013). It is likely that FTS is much more readily released from PEG_5K_-FTS_2_(L) than PEG_5K_-FTS_2_(S) with a stable amide linkage. *In vitro*, PEG_5K_-FTS_2_(L) alone was much more active than PEG_5K_-FTS_2_(S) in antitumor activity in both 4T1.2 and HCT-116 cell lines. *In vivo*, PTX formulated in PEG_5K_-FTS_2_(L) showed antitumor activity that is more potent than that of Taxol or PTX/PEG_5K_-FTS_2_(S). Glutathione is present in tumors at much higher concentrations than in extracellular fluids (Saito et al., 2003; Zhang et al., 2018b), so coupling the FTS to PEG *via* a disulfide linkage can lead to greater release of FTS inside tumor cells and therefore to greater cytotoxicity than either Taxol or PTX (Zhang et al., 2014g).

Screening of two weights of PEG (PEG_2K_ *vs.* PEG_5K_) and two molar ratios PEG: FTS (1:2 or 1:4) identified PEG_5K_-FTS_4_ as the stablest and most cytotoxic micelles against 4T1.2 breast cancer tumors in mice (Zhang et al., 2014e). Adding the 9-fluorenylmethoxycarbony (Fmoc) moiety to these micelles significantly improved the loading capacity of PTX and DOX as well as efficacy against breast and prostate cancer tumors in mice (Zhang et al., 2014f). Incorporating a disulfide linkage into Fmoc-modified PEG_5K_-FTS_2_ micelles reduced the CMC to 0.02 μM, which was 10-fold lower than the CMC of micelles without either the Fmoc moiety or disulfide linkage (Xu et al., 2017). It also increased accumulation inside prostate cancer tumors in mice by two- to three-fold. The disulfide linkage also increased the cytotoxicity *in vitro* and *in vivo* when Fmoc-modified micelles were loaded with PTX. These studies indicate that PEG-FTS is an effective dual-functional carrier that can synergize with co-delivered anti-cancer agents.

### PEG-Derivatized Anticancer Drugs

Most small-molecule anti-cancer drugs are hydrophobic. Their water solubility and circulation time can be improved by creating PEGylated prodrug copolymers that self-assemble into micelles. These PEG-drug conjugates can also be used to carry additional chemotherapeutic agents (**Table 1**). Below, we discuss only PEGylated prodrug complexes to which additional drugs are added. PEGylated drug complexes without further drug addiction have been reviewed elsewhere.

**Table 1:** Polyethylene glycol (PEG)-derivatized anticancer drug as dual-functional micellar carrier.

Self-assembled prodrugs	Hydrophobic drug	Co-loaded drug	*In vitro*/*in vivo* experimental model	References
CPT-SS-PEG-SS-CPT	Camptothecin	Camptothecin	HepG2 human hepatoma cell model	(Li et al., 2011)
CPT-PEG-CPT	Camptothecin	Doxorubicin	Human cervical cancer Hela cells	(Dong et al., 2012)
mPEG-BC-PGluCPT	Camptothecin	Doxorubicin	HepG2 and HL-7702 cells	(Gao et al., 2018)
P(CPTMA-coPEMA)	Camptothecin	Glucose oxidase	A549 tumors	(Li et al., 2017)
PEG-bPTCPT	Camptothecin	β-lapachone	Mouse breast cancer 4T1 cell line and 4T1 tumor-bearing mice	(Yin et al., 2019)
PEG-PTX	Paclitaxel	Paclitaxel	MCF-7 cells and mouse xenograft tumor model bearing MCF-7 cells	(Lu et al., 2014a)
PEG-acetal-PTX	Paclitaxel	Paclitaxel	HeLa and MDA-MB-231 cells	(Huang et al., 2018)
mPEG-b-DOX	Doxorubicin	Verapamil	MCF-7/ADR cell line	(Zhao et al., 2015)
PEG-CH = N-DOX	Doxorubicin	7-ethyl-10-hydroxyl-camptothecin	MCF-7 cells and mouse xenograft tumor model bearing MCF-7 cells	(Sun et al., 2018b)
PEG–DTX	Docetaxel	Docetaxel	H460 human non-small cell lung, SKOV-3 human ovarian and MCF-7 human breast cancer cells	(Liu et al., 2008)
PEG-CCM@APTES-COF-1	Curcumin	Doxorubicin	HeLa cells and HeLa cell xenograft tumor mice model	(Zhang et al., 2018a)
PEG-Fmoc-GA	Glycyrrhizic acid	Doxorubicin	HepG2 cells human liver cancer xenograft model	(Yang et al., 2018b)

The natural cytotoxic quinolone alkaloid CPT inhibits DNA topoisomerase I, making it effective against a wide spectrum of cancers (Gromova et al., 1990; Pizzolato and Saltz, 2003; Zunino and Pratesi, 2004). However, the clinical use of CPT is limited by its extremely hydrophobicity and low stability *in vivo* (Koo et al., 2005; Botella and Rivero-Buceta, 2017). A CPT-PEG-CPT prodrug has been prepared by conjugating two molecules of CPT to the ends of PEG *via* degradable ester linkage (Dong et al., 2012). This complex, which can accommodate CPT up to 41%, self-assembled into micelles with a diameter of 50 nm. DOX was loaded into the micelles with high capacity, and the complex showed greater cytotoxicity than free DOX. The increased cytotoxicity might be induced by the combined effect of both DOX and CPT. Further, they introduced redox-responsive disulfide linkers and prepared a micellar system (CPT-SS-PEG-CPT) to achieve an aim of rapidly releasing the native CPT in tumor-relevant reductive conditions (Li et al., 2011). An amphiphilic prodrug of CPT has been prepared by polymerizing γ-CPT glutamate N-carboxyanhydride with mPEG *via* pH-sensitive boronate ester bonds (Gao et al., 2018). The resulting prodrug self-assembled into micelles, simultaneously encapsulating DOX. These micelles showed sustained drug release during 48 h, which increased when pH was reduced from 7.4 to 6.0. This approach may promote uptake of the micelles and drug release specifically in the acidic tumor microenvironment.

PTX, isolated from *Taxus brevifolia*, is one of the most effective chemotherapeutic drugs against ovarian, lung, breast, and other cancers (Wani et al., 1971; Blair and Deeks, 2015; Bourgeois-Daigneault et al., 2016). Its aqueous solubility is only about 0.3 mg/ml, so it is solubilized with polyoxyethylenated castor oil (Cremophor EL) in the commercial formulation of PTX, called Taxol. Cremophor EL can cause serious hypersensitivity, neurotoxicity, and nephrotoxicity, which severely limit the success of PTX chemotherapy (Singla et al., 2002; Nehate et al., 2014). To avoid these problems, mPEG5000 and PTX have been conjugated *via* succinic anhydride as linker, giving a water-soluble PTX prodrug (Greenwald et al., 1996). Although this prodrug appears to have negligible anti-tumor activity because PTX release is too low (Liang et al., 2012), the prodrug can complex with free PTX to form spherical particles of 100–200 nm (Lu et al., 2014a). These particles inhibited the growth of MCF-7 tumors in mice significantly better than Taxol, and with less toxicity. To allow breakage of the PEG-PTX bond under certain conditions, PEG and PTX have been conjugated using an acetal linker that cleaves preferentially when exposed to endosomal pH (Huang et al., 2018). This prodrug self-assembles into micelles and can encapsulate free PTX into the hydrophobic core, such that the total PTX concentration in the micelles reaches a capacity of 60.3%. These PTX-loaded micelles inhibited HeLa and MDA-MB-231 cells more than free PTX.

## PEG-Derivatized Dual-Functional Micelles for Immunochemo-Therapy

Accumulation evidence suggests that chemotherapy-induced immune responses contribute to the overall anti-tumor effects of chemotherapy (Vanneman and Dranoff, 2012; Bracci et al., 2014; Kumar et al., 2017). However, cancer treatment also up-regulates various negative feedback mechanisms that limit the effectiveness of chemotherapy-elicited immune responses as well as other immunotherapies (Curran et al., 2010; Jiang et al., 2019). For example, indoleamine 2,3-dioxygenase (IDO) is a negative feedback protein that helps tumors evade the immune system, grow, and metastasize (Soliman et al., 2010; Ricciuti et al., 2019; Zhu et al., 2019); IDO causes immunosuppressive effects by inhibiting T cell differentiation and proliferation (Uyttenhove et al., 2003; Zamanakou et al., 2007). Reversing these negative feedback mechanisms may have anti-tumor effects. NLG919, a highly selective IDO inhibitor with a median effect concentration of 75 nM, promotes growth of T and natural killer cells, increases interferon production, and reduces conversion to a regulatory T cell phenotype (Mautino et al., 2013; Li et al., 2014; Meng et al., 2017). NLG919 is poorly water soluble, and it cannot be simply co-delivered as a mixture with drugs because of differences in physico-chemical and pharmacokinetic profiles (Liu et al., 2010).

NLG919 has been conjugated with PEG in a dual-functional, immunostimulatory micelle in which the Fmoc group was introduced to improve drug loading capacity and formulation stability (Chen et al., 2016). The resulted PEG_2K_-Fmoc-NLG micelles self-assembled in aqueous solutions to form particles of about 90 nm with a CMC of 0.737 mM. It’s found that PEG_2K_-Fmoc-NLG with a relatively labile ester linkage well retained the pharmacological activity of NLG919 and significantly enhanced T-cell immune responses. The micelles could also be loaded with several common drugs: PTX, docetaxel, DOX, gefitinib, imatinib, and curcumin. PTX-loaded PEG_2K_-Fmoc-NLG micelles were delivered intravenously into mice bearing breast cancer tumors or melanomas, and they showed significantly greater anti-tumor response than free PTX or the empty micelles without PTX.

These PTX-loaded micelles also enhanced anti-tumor immune responses, yet they triggered the counter-productive accumulation of myeloid-derived suppressor cells (MDSCs), which maintain a highly immunosuppressive tumor microenvironment (Pastaki Khoshbin et al., 2019; Safari et al., 2019; Groth et al., 2019). MDSCs can induce immunosuppression by up-regulating inducible nitric oxide synthase and arginase I, thereby inhibiting T-cell function (Meirow et al., 2015). MDSCs can enhance tumor cell proliferation, facilitate tumor metastasis and angiogenesis, and promote drug resistance (Draghiciu et al., 2015; Gabrilovich, 2017). Increased numbers of tumor-infiltrating MDSCs often correlate with high tumor burden and metastasis, contributing to poor prognosis (Nagaraj and Gabrilovich, 2008; Malek et al., 2016). To avoid stimulating MDSCs, a dual-functional carrier of PEG linked to NLG919 *via* an Fmoc moiety was loaded with both PTX and sunitinib (Chen et al., 2018), an FDA-approved tyrosine kinase inhibitor for the treatment of various cancers. Sunitinib reduces the level and activity of MDSCs and Treg cells systemically and in the tumor microenviroment (van Hooren et al., 2016; Motzer et al., 2017). These micelles then deliver three anti-tumor components to the cancer: PTX and sunitinib lead to synergistic killing of tumor cells as well as the induction of anti-tumor immune responses following the release and presentation of tumor antigens, sunitinib inhibits cytokines needed to recruit MDSCs, and NLG919 is released quite slowly to inhibit IDO and thereby sustain an immune-active tumor microenvironment.

In other studies, the dual-functional carrier of PEG-linked to NLG919 *via* an Fmoc moiety was loaded with DOX for immunochemotherapy against lymphoma (Zhai et al., 2017). The DOX-loaded micelles were small (<120 nm), showed sustained drug release, and triggered significantly large numbers of total CD4+/CD8+ T cells and functional CD4+/CD8+ T cells in mice with lymphoma than the same micelles without DOX. These micelles, whether loaded with DOX or not, reduced the recruitment of granulocytic MDSC and monocytic MDSCs to tumor tissues. This contrasts with the observation that the same micelles, when loaded with PTX, triggered the counter-productive accumulation of MDSCs (Chen et al., 2018). These differences may reflect the different abilities of DOX and PTX to modulate the immune system. More studies are needed to better understand the impact of different immunochemotherapies on the tumor microenvironment and overall anti-tumor effects.

## PEG-Derivatized Dual-Functional Micelles With Improved Drug Loading Capacity

Dual-functional micelles not only exert intrinsic therapeutic effects, but also deliver other drugs to tumor sites. Most of these micelles load drugs *via* hydrophobic interaction, hydrogen bonding, and π-π stacking between the encapsulated drug and the hydrophobic components of the micelle (Lu et al., 2013a; Mohamed et al., 2014). This means that one way to improve drug loading capacity is to increase the density of the hydrophobic component. Conjugating PEG to multiple vitamin E molecules rather than one molecule improved drug loading capacity (Wang et al., 2012), and increasing the number of vitamin E, embelin, or FTS molecules conjugated to one PEG molecule increased drug loading capacity (Lu et al., 2013b; Lu et al., 2014c; Zhang et al., 2014e). We found that conjugating PEG to six vitamin E molecules (PAMV_6_) gave the highest drug loading capacity (Li et al., 2016b). The wheat-like structure of the hydrophobic chains of PAMV_6_ likely creates a binding pocket that enhances the interaction between carrier and loaded drug. However, increasing the number of conjugated vitamin E molecules to 10 (PAMV_10_) gave heterogeneous micelles, presumably because the balance between hydrophilic and hydrophobic chains was destroyed. These results suggest that increasing the density of the hydrophobic component can increase drug loading capacity only to a certain extent.

While the dual-functional micelles discussed so far in this review are effective in formulating drugs that are strongly hydrophobic, they are less effective at formulating moderately hydrophobic drugs. This can be improved by introducing hydrotropic motifs into the hydrohobic domain of polymeric micelles, which improves drug loading capacity and micelle stability (Cho et al., 2004; Huh et al., 2005). In fact, covalent coupling of the moderately hydrophobic DOX into the hydrophobic domain of polymeric micelles improves the loading capacity of free DOX (Yoo and Park, 2004b; Yoo and Park, 2004a).

Another way to improve drug-loading capacity and formulation stability is to incorporate a surfactant at the interface between carrier and drug (Gao et al., 2013). The Fmoc moiety, a functional group routinely used to protect amino acids, is one of the most potent drug-interacting motifs (**Table 2**). Fmoc-based micelles can effectively encapsulate six drugs with diverse structures: probucol, niclosamide, progesterone, cyclosporin A, nifedipine, and griseofulvin (Gao et al., 2013; Zhang et al., 2014b). Hydrophobic interactions and π-π stacking may contribute to drug-Fmoc interactions. Consistent with this idea, introduction of the Fmoc domain into PEG-derivatized vitamin E (Zhang et al., 2014f; Xu et al., 2017) and FTS carriers (Zhang et al., 2014i; Lu et al., 2015) improves their drug-loading capacity, probably because the Fmoc can engage in stacking interactions with the interfacial aromatic ring of vitamin E and FTS. A PEG-derivatized ibuprofen conjugate (PEG_2K_-FIbu) consisting of a hydrophilic PEG segment, Fmoc motif, and hydrophobic ibuprofen domain readily formed stable micelles with PTX in the nanosize range (∼100 nm) to a relatively high loading capacity of 67% (Zhao et al., 2016). *In vitro* cytotoxicity showed that PTX formulated in PEG_2K_-FIbu micelles was comparable to Taxol in inhibiting the proliferation of tumor cells. The lack of synergy between the ibuprofen-based carrier and PTX is likely due to the very low concentrations of carriers used in the cytotoxicity study. *In vivo*, PTX-loaded PEG_2K_-FIbu micelles showed a much more pronounced therapeutic efficacy compared with Taxol formulation. The improved antitumor activity of PTX-loaded PEG_2K_-FIbu micelles is likely attributed to the enhanced delivery of PTX to the tumors and the potential synergy between the micellar carrier and PTX.

**Table 2:** Fmoc-based PEG-derivatized dual-functional micellar systems.

PEG-derivatized micellar carriers	Hydrophobic segment	Co-loaded drug	Drug loading capacity	Drug loading efficiency	References
PEG_5K_-Fmoc-VE_2_	Vitamin E	Doxorubicin	39.9%	79.5%	(Lu et al., 2014c)
PEG_5K_-(Fmoc-VE)_2_	Vitamin E	Paclitaxel	20.8%	80.7%	(Zhang et al., 2014i)
PEG_5K_-Fmoc-VE_2_	Vitamin E	Camptothecin	9.2%	70.5%	(Lu et al., 2015)
PEG_5K_-Fmoc-FTS_2_	S-trans,transfarnesylthiosalicylic acid	Paclitaxel Doxorubicin	12.1% 32.8%	55.4% 69.7%	(Zhang et al., 2014f)
PEG_5K_-Fmoc-S-S-FTS_2_	S-trans,transfarnesylthiosalicylic acid	Paclitaxel	34.0%	53.5%	(Xu et al., 2017)
PEG-Fmoc-GA	Glycyrrhizic acid	Doxorubicin	7.3%	93.2%	(Yang et al., 2018b)
PEG_2K_-Fmoc-NLG	NLG919	Paclitaxel	24.7%	–	(Chen et al., 2016)
PEG_2K_-Fmoc-NLG	NLG919	Paclitaxel	5.6%	98.3%	
		Sunitinib	8.0%	96.4%	(Chen et al., 2018)
PEG_2K_-Fmoc-NLG	NLG919	Doxorubicin	15.6%	93.6%	(Zhai et al., 2017)
PEG_2K_-Fmoc-Ibu	Ibuprofen	Paclitaxel	67.3%	88.4%	(Zhao et al., 2016)

## Challenges and Future Considerations of Dual-Functional Micellar Carrier

A large number of preclinical studies on dual-functional micelles have been published, which showed great promise to be an effective nanomedicine platform for drug delivery and cancer therapy. However, the clinical translation and desired therapeutic effects of dual-functional micelles are still far from satisfactory (Cabral and Kataoka, 2014). Micellar system share the limitations inherent to other nanodelivery system which mainly rely on the passive targeting of tumors based on the EPR effect (Fang et al., 2011). Poor tumor penetration due to high interstitial pressure and inefficient tumor cell uptake as well as off target liver and spleen accumulations have been repeatedly reported with the use of micelles (Taurin et al., 2012). The targeting effect in human sometimes is not as pronounced as that obtained in the simple cell or animal models due to the complexity of the human body. To this end, ligand-installed polymeric micelles have the potential for overcoming such biological barriers by taking advantage of receptor-mediated active target mechanisms (Deshayes et al., 2013). The end-group of the PEG segment can be readily modified with ligands capable of recognition of cell-specific surface receptors, providing cellular selectivity and superior intracellular delivery to dual-functional polymeric micelles. The effect of various ligands, including peptides, antibody fragments, and small molecules, installed on nanocarriers, has been confirmed to improve their targeting efficiency (Allen, 2002; Bae et al., 2005). For example, cyclic-RGD (cRGD) peptides have been recognized as specific ligands for integrin αvβ3 which is overexpressed in neovasculature and tumor cells (Kulhari et al., 2015; Li et al., 2019). cRGD-modified polymeric micelles loaded with DACHPt not only enhanced the cellular uptake of micelles by cancer cells, but also achieved efficient drug delivery in a mouse model of glioblastoma (Miura et al., 2013). Even though glioblastoma is notorious for its poor permeability due to the presence of the blood–brain tumor barrier, these cRGD-installed micelles rapidly penetrated and accumulated within tumor tissues.

Besides, the premature drug release in the bloodstream and inadequate drug release in the tumor may also account for the inferior therapeutic effects in human patients. Most of toxicities reported in clinical trials resulted from the micelle releasing the drug in the blood, causing toxic side effects similar to the free drug and thereby deviate from tumor targeting (Miller et al., 2013). Ideally, the drug is retained in the polymeric micelles during circulation but is released after accumulation in the tumor interstitium. The design of stimuli-responsive nanomicellar carriers is a very effective way to achieve tumor-targeted drug delivery and adequate release (Zhou et al., 2018a). Numerous stimuli-responsive dual-functional nanomicelles have been developed to deliver anticancer drug in response to a variety of extra/intracellular stimuli or external triggers, including pH, redox potential, enzymes, temperature, light, ultrasound, and magnetic field (Kalhapure and Renukuntla, 2018). It is reported that the microenvironment of most solid tumors is intrinsically acidic (pH 6.5–7.2), while the pH value in the blood and normal tissue is about 7.4. An even lower acidic pH is found in endosomes and lysosomes (pH 4.5–6.5) (Qu et al., 2017a; Liang et al., 2019). Accordingly, the difference in pH has been widely exploited to achieve tumor site- or organelle-specific activation of pH-responsive nanomicelles. Yang et al. developed a novel multifunctional pH-sensitive nanomicelle based on luteinizing hormone-releasing hormone (LHRH)-PEG-PHis-DOX/DOX-trans-activating transcriptional activator (TAT) acid-sensitive micelle, which could dissociate and release DOX-TAT when responding to the tumor extracellular pH (Yang et al., 2015). Such systems could efficiently go across the cell membrane and reach the cytosol of the multidrug-resistant cancer cells, resulting in the remarkable antitumor efficacy and negligible systematic toxicity.

In addition, PEG is believed to provide a steric barrier around the nanocarriers, and thus prolonging their blood circulation time. However, there was an unexpected pharmacokinetic alteration brought about with a second dose of PEGylated nanoparticles, the so-called accelerated blood clearance (ABC) phenomenon (Ishida and Kiwada, 2008). With this phenomenon, a second dose of PEG-derivatized nanomicelles, PEGylated liposomes, or PEG-containing microemulsions is rapidly cleared from circulation when administered within a certain time interval from injection of the first dose due to an enhanced accumulation in the liver (Abu Lila et al., 2013). Many attempts have focused on the use of other polymers, such as chitosan and hyaluronic acid, to replace PEG modification and to avoid the occurrence of the ABC phenomenon (Dong et al., 2016). Moreover, polyetherimide-based dual-functional carrier is an alternative strategy for the delivery of charged macromolecules, such as antisense oligonucleotides, plasmid DNA, and siRNA/miRNA (Zhou et al., 2018b). David Oupicky’s group developed many polycationic CXCR4 antagonist based dual-functional nanomicellar carriers that can simultaneously deliver siRNA and inhibit CXCR4 to achieve combination anticancer therapy (Li et al., 2012; Zhou et al., 2018b). Such dual-function delivery vectors could enhance antimetastatic efficacy of a variety of cancer gene therapy methods (Li and Oupicky, 2014; Sun et al., 2018a).

## Conclusions

Dual-functional nanomicelles have emerged as a versatile and powerful platform for cancer therapy. They can self-assemble and efficiently encapsulate chemotherapeutic agents such as PTX and DOX into their hydrophobic core. They exert anti-tumor effects by virtue of the encapsulated drug as well as the intrinsic anti-cancer activity of carrier materials in the micelles. As a result, these micelle systems can enhance the efficacy of anti-tumor drugs while reducing their systemic toxicity; in fact, the carrier materials and drug cargo can synergize to increase efficacy.

Most of these dual-functional micelles rely on passive targeting, so more effective strategies are needed to enhance their targeted delivery, deepen their penetration into the tumor, and enable drug release in response to specific stimuli. Much work remains to be done to translate these promising nanomaterials into the clinic, including scale-up for industrial production and validation studies in appropriate animal models.

## Author Contributions

YL and JH wrote the manuscript and obtained funding, TZ and QL contributed to the discussion and review of the manuscript.

## Funding

This work was financially supported by the National Natural Science Foundation of China (81603035 and 81870599), China Postdoctoral Fellowship (2017M612981 and 2018T110986).

## Conflict of Interest Statement

The authors declare that the research was conducted in the absence of any commercial or financial relationships that could be construed as a potential conflict of interest.
